# Screening of Microbial Natural Products and Biological Evaluation of Trichomicin as Potential Anti-Cytokine Storm Agents

**DOI:** 10.3389/fphar.2021.770910

**Published:** 2021-12-09

**Authors:** Yu Chen, Zhuochen Zhuang, Jing Yang, Liping Bai

**Affiliations:** ^1^ School of Basic Medicine and Forensic Medicine, Baotou Medical College, Baotou, China; ^2^ NHC Key Laboratory of Biotechnology of Antibiotics, CAMS Key Laboratory of Synthetic Biology for Drug Innovation, Institute of Medicinal Biotechnology, Chinese Academy of Medical Sciences & Peking Union Medical College, Beijing, China

**Keywords:** cytokine storm, Trichomicin, microbial natural products, LPS, COVID-19

## Abstract

COVID-19 has remained an uncontained, worldwide pandemic. Most of the infected people had mild symptoms in the early stage, and suddenly worsened or even died in the later stage which made the cytokine release syndrome (CRS) once again aroused people’s attention. CRS is an excessive immunity of the body to external stimuli such as viruses, bacteria, and nanomaterials, which can cause tissue damage, local necrosis or even death. Lipopolysaccharide (LPS) is one of the most effective CRS inducers, which can activate macrophages to release cytokines, including tumor necrosis factor (TNF-α), interleukin-1β (IL-1β), IL- 6 and chemokines. We used RT-PCR to detect the expression of representative cytokines in mouse and human cells at different concentrations of Trichomicin, Ebosin, and 1487B after LPS stimulation. The results showed that the expression of TNF-α, IL-1β, IL-6, and CXCL10 all increased after LPS stimulation. Among the various drugs, Trichomicin had the most obvious inhibitory effect on cytokine expression *in vitro*, and it was further verified *in vivo* that Trichomicin can improve the survival rate of mice stimulated with LPS. Finally, it was proved that Trichomicin inhibited the Stat3 and NF-κB pathways and reduced the phosphorylation of Stat3 and p65 after LPS stimulation, thereby inhibiting the response of macrophages to pro-inflammatory stimuli. The article clarified the inhibitory activity and mechanism of action of Trichomicin on CRS, and laid the foundation for the research on the anti-cytokine storm activity of microbial natural products.

## Introduction

Coronavirus disease 19 (COVID-19) is caused by severe acute respiratory syndrome coronavirus 2 (SARS-CoV-2). WHO declared a COVID-19 pandemic in January 2020. The virus rapidly spread across the world through travelers and the number of world-wide cases has exceeded 72 million with over one million deaths as of December 14, 2020 (Chung et al., 2021). Most of patients have mild symptoms, but a significant proportion of patients have developed a severe acute hyperimmune response characterized by cytokine storm (Caricchio et al., 2021). It includes systemic hyperinflammation, acute respiratory distress syndrome (ARDS) and multiorgan failure. In fact, the ultimate culprit is not caused by the virus itself, but the body’s own immune system that the virus intervenes, and its excessive immune response brews into a cytokine storm, which causes the dysfunction of the body’s organs. This is one of the important reasons that cause the sudden increase in the condition of the new crown infection and even the death (Shinya et al., 2012; Casadevall and Pirofski, 2014).

Cytokine storm, also known as cytokine release syndrome (CRS), is an excessive and uncontrollable release of inflammatory cytokines and chemokines, leading to a self-balancing detrimental process, and is considered to be a major cause of COVID-19 disease severity (Coperchini et al., 2021). It has been shown that several cytokines such as IL-6 and IL-1 are involved in the severity of COVID-19 disease (Kim et al., 2021). The elevated serum levels of IL-6 in COVID-19 patients and its circulating levels are positively correlated with the severity of disease, indicating that IL-6 plays a key role in the pathogenesis of CRS (McGonagle et al., 2020; Ragab et al., 2020; Zhang et al., 2020).

In CRS, the three most important cytokines in the IL-1 family are IL-1β, IL-18 and IL-33, in which IL-1β cytokine has been studied the most (Ye et al., 2020). IL-1β plays a pro-inflammatory role in recruiting immune cells and inducing secondary cytokine production, leading to an acute phase response. Coperchini et al. proposed that COVID-19 patients have high levels of the pro-inflammatory cytokines IL-1β, IL-6, TNF-α, INF-γ and chemokines CXCL10, CXCL9 levels (Coperchini et al., 2020). In addition, circulating levels of CXCL10 are elevated at admission and remain high during disease progression (Coperchini et al., 2021). Various cytokines promote or inhibit the expression of each other in the body, forming a complicated cytokine regulatory network (Zhang et al., 2013a). However, when body is severely infected, the balance between immune-promoting and immune-inhibiting mechanisms promoted by cytokines will be broken, which may result in CRS, a leading cause of death of COVID-19 patients (Ragab et al., 2020). Therefore, suppression of CRS can effectively prevent the aggravation of COVID-19 patients.

At present, there is no specific treatment for CRS and ARDS in clinical practice, and treatment measures such as administration of anti-infective drugs, glucocorticoids, and artificial ventilation assistance are mostly used. IL-6 antagonists such as tocilizumab and stutuzumab can block downstream signal transduction through receptor binding, effectively controlling CRS without affecting the efficacy of CAR-T cells (Maude et al., 2014; Wang et al., 2018a). Glucocorticoids can combine with corticosteroid binding protein and albumin to form a complex, and a small amount of free hormones diffused through the cell membrane to bind to the glucocorticoid receptor in the cytoplasm, and enters the nucleus to induce or inhibit the expression of inflammation-related genes and exert anti-inflammatory effects (Zhang and Jiang, 2015). Cytokine blockers such as Etanercept and Anakinra can also bind to their targeting cytokines to inhibit CRS (Zou et al., 2017; Giavridis et al., 2018; Norelli et al., 2018). In addition, catecholamine modulators such as atrial natriuretic peptide and α-methyltyrosine (Staedtke et al., 2018), sphingosine analogs such as Siponimod (Zhang et al., 2013a), Ulinastatin (Tao et al., 2017) and the plasma of recovered patients also show varying therapeutic potential for CRS. However, among the above-mentioned drugs, cytokine blockers have single target, and glucocorticoids may cause side effects such as double infection and diabetes, osteoporosis and high blood pressure. Therefore, the development of new drugs that effectively inhibit CRS has become an urgent need.

Ebosin is a novel exopolysaccharide (EPS) isolated from the fermentation culture of *Streptomyces* sp. 139 (Zhang et al., 2016). Preliminary pharmacodynamic research showed that Ebosin has significant therapeutic effect in rat type II collagen-induced arthritis (CIA) models and adjuvant rheumatoid arthritis animal models. Following its treatment, the expression level of IL-1β and TNF-α decreased significantly *in vivo*, indicating that the inhibitory effect of Ebosin on rheumatoid arthritis may be attributed to the reduced levels of related inflammatory cytokines (Zhang et al., 2013b; Zhang et al., 2016). Trichomicin is a small molecule compound with new structure isolated from *Trichoderma harzianum* (*T. harzianum*) in our laboratory (Zhu et al., 2020). Early *in vitro* studies confirmed that Trichomicin has significant anti-inflammatory and anti-tumor activities, and inhibits the growth of colon tumor (Qi et al., 2011; Zhao et al., 2020). Furthermore, our laboratory also used the anti-inflammatory model to screen the new structure small molecule compound 1487B. Pharmacodynamic studies showed that 1487B has a significant inhibitory effect on the acute inflammation model of mouse ear swelling (Ma et al., 2015). The above results suggest that Ebosin, Trichomicin and 1487B may have anti-inflammatory activities, and we speculate that they may also have an inhibitory effect on CRS.

Lipopolysaccharide (LPS) is one of the most effective CRS inducers. It can activate monocytes/macrophages to release cytokines, including tumor necrosis factor (TNF-α), interleukin-1β (IL-1β), IL-6, chemokines, and other inflammatory cytokines (Cavaillon, 2018). Not only that, LPS can induce sepsis and toxic shock syndrome in mice. Therefore, relevant research always uses LPS to induce the release of pro-inflammatory cytokines in both *in vivo* and *in vitro* models. In this study, we used LPS to stimulate cells or mice, and used RT-PCR and western blot methods to conduct a preliminary study to compare the anti-cytokine storm activities of Trichomicin, Ebosin and 1487B, and further discuss their possible mechanisms of action. This study aims to lay the foundation for the research on the anti-cytokine storm activity of a variety of microbial natural products.

## Materials and Methods

### Materials

Ebosin (50 mg/ml), 1487B (20 mM) and Lipopolysaccharide (LPS, 1 mg/ml, Beyotime, China) were dissolved in Phosphate-Buffered Saline (PBS, Corning, Manassas, United States). Filtered through a 0.22 μm filter, they were stored at −80°C until use. Trichomicin (4 mM) and PMA (10 mM, Sigma, GER) were dissolved in DMSO, and were further diluted with medium (for cell assays). Sodium carboxymethyl cellulose (CMC-Na, Selleckchem, United States) was dissolved in double distilled water at 0.5 mg/ml and used as a solvent for animal experiments.

### Cell Lines and Culture

RAW264.7, NR8383, J774A.1 and THP-1 cells were purchased from Cell Resource Center, Institute of Basic Medical Sciences, Chinese Academy of Medical Sciences (CAMS, Beijing, China). U937 and HL-60 cells were derived from Beijing BeNa Culture Collection (BNCC) Biotechnology Company. RAW, J774A.1 and NR8383 cells were conventionally cultivated in Dulbecco ҆s modified Eagle medium (DMEM) with high glucose (Corning, Manassas, United States). THP-1 and U937 cells were routinely cultivated in RPMI 1640 medium (Corning, Manassas, United States). HL-60 cells were routinely cultivated in Iscove’s modified Dubecco’s medium (IMDM, Corning, Manassas, United States). The media were supplemented with 10% fetal bovine serum (FBS, Invitrogen, Carlsbad, CA, United States) and antibiotics (penicillin and streptomycin, 100 U/mL) (Corning, Manassas, United States) under standard conditions (37°C, 5% CO_2_).

To generate macrophage-like differentiated cells, THP-1, U937 and NR8383 cells were incubated with 160 nM PMA, HL-60 cells were incubated with 1.25% DMSO at 37°C for 24 h (Aldo et al., 2013). After incubation, the PMA/DMSO-containing medium was removed and replaced with medium for subsequent experiments.

### Experimental Animals

Female BALB/c mice of bodyweight 18–22 g were purchased from Beijing Huafukang Biotechnology Co., Ltd., allowed to acclimate to a new SPF surrounding (temperature: 22 ± 2°C, humidity: 40–60%, light/dark cycle: 12 h) for 1 week, with food and water supplied ad libitum. Animal experimental protocols were performed under NO. IMB-20210601D_7_01 according to the Chinese National Guidelines for the Care and Use of Laboratory Animals and approved by the Animal Experimental Ethics Committee of Institute of Medicinal Biotechnology, Chinese Academy of Medical Sciences & Peking Union Medical College.

### Cell Viability Assay

The sensitivities of the cell lines RAW, NR8383, THP-1 and HL-60 to Trichomicin, Ebosin and 1487B compounds were evaluated by MTT assay. Each cell line was seeded in a 96-well plate. After 24 h incubation, the medium was changed and Trichomicin/Ebosin/1487B was added to cell culture medium with different concentrations. The cells were cultured for 72 h, supplemented with 10 μl MTT (Boster, Wuhan, China) solution, and incubated for another 4 h, after which the medium was removed and 100 μl of DMSO was added. Finally, the absorbance at 570 nm of each well was measured with a Victor X5 multi-label microplate detector (PerkinElmer, MA, United States).

### RNA Purification, cDNA Synthesis, and Real-Time Reverse Transcription PCR (RT-PCR)

Total mRNA was extracted from cells with TRIzol Reagents (TransGen Biotech, Beijing, China), and was transcribed into cDNA with TransScript One-Step gDNA Removal and cDNA Synthesis SuperMix according to the manufacturer’s instructions. The amplification protocol consists of an initial step of 30 s at 94°C followed by 45 cycles of 5 s at 94°C and 30 s at 60°C on a Bio-Rad CFX96 (Bio-Rad, United States) using PerfectStart Green qPCR SuperMix (TransGen Biotech, Beijing, China). The relative expression levels were normalized to gapdh level using the 2^−∆∆CT^ method. The primer sequences are listed in Table 1.

**TABLE 1:** The primers used for RT-PCR.

Gene name	Forward primer (5′-3′)	Reverse primer (5′-3′)
*human il-1β*	CTG​TCC​TGC​GTG​TTG​AAA​GA	TTC​TGC​TTG​AGA​GGT​GCT​GA
*human tnf-α*	TGT​AGC​AAA​CCC​TCA​AGC​TG	TTG​ATG​GCA​GAG​AGG​AGG​TT
*human-il-6*	CCA​CAC​AGA​CAG​CCA​CTC​AC	TTT​CAC​CAG​GCA​AGT​CTC​CT
*human cxcl10*	TCT​AAG​TGG​CAT​TCA​AGG​AGT​ACC	GGA​CAA​AAT​TGG​CTT​GCA​GGA
*human-gapdh*	GGA​GCG​AGA​TCC​CTC​CAA​AAT	GGC​TGT​TGT​CAT​ACT​TCT​CAT​GG
*mouse-il-1β*	TGC​CAC​CTT​TTG​ACA​GTG​AT	AAG​GTC​CAC​GGG​AAA​GAC​AC
*mouse-tnfα*	GTC​CCC​AAA​GGG​ATG​AGA​AGT	TTT​GCT​ACG​ACG​TGG​GCT​AC
*mouse-il-6*	AGT​TGC​CTT​CTT​GGG​ACT​GA	CAG​AAT​TGC​CAT​TGC​ACA​AC
*mouse-cxcl10*	GTC​TGA​GTG​GGA​CTC​AAG​GGA​T	AGG​CTC​GCA​GGG​ATG​ATT​TC
*mouse-gapdh*	AAC​AGC​AAC​TCC​CAC​TCT​TC	CCT​GTT​GCT​GTA​GCC​GTA​TT

### Western Blot

Total protein samples of cells were prepared by RIPA buffer (Boster, China) with phosphatase inhibitor (1:1,000, Applygen, Beijing, China) on ice-bath for 1 h. The supernatant was collected by centrifugation at 14,000 rpm for 20 min at 4°C, and the protein concentration was measured by a BCA protein assay kit (Applygen, Beijing, China). The protein samples were loaded to an 10% SDS-PAGE gel and was transferred to a PVDF membrane. The PVDF membranes were blocked with 5% (w/v) skimmed milk powder in TBST for 2 h, and were subsequently incubated with primary anti-phospho-NF-κB p65 (#3033, Cell Signaling Technology, Boston, United States), anti-NF-κB p65 (#8242, Cell Signaling Technology, Boston, United States), anti phospho-Stat3 (#9145S, Cell Signaling Technology, Boston, United States), anti Stat3 (#JA9179, Calbiochem, NJ, United States), anti-phospho-IKKα/β (#2697, Cell Signaling Technology, Boston, United States), anti-IKKβ (#8943, Cell Signaling Technology, Boston, United States) and anti GAPDH (AMM04703G, Santa Cruz Biotechnology, Beijing, China) at 4°C overnight. Then the membranes were washed with TBST and were incubated with secondary HRP-conjugated goat anti-rabbit or antimouse IgG antibody (1:10,000) for 2 h at room temperature. The blots were detected with an enhanced chemiluminescence method on a Bio-Rad Gel imaging system (733BR-2008, Bio-Rad, CA, United States) and were analyzed by ImageJ software.

### Model and Animal Treatment

In the LPS-induced inflammation model, mice were randomly divided into four groups (*n* = 8): placebo (CMC-Na) group, LPS only model group (4 mg/kg), LPS with Trichomicin low dose (15 mg/kg) and high dose (30 mg/kg) treatment groups. Placebo and Trichomicin groups were applied intragastrically from day 1 to day 14. At day 8, mice within the model group and treatment group were intraperitoneally injected with LPS (4 mg/kg) 1 h after administration to establish the model. The changes in the weight of mice were monitored and their survival were observed every day. All mice were euthanized at day 14.

### Statistical Analysis

All data were analyzed using Graph Pad Prism 8.4.2 (Graph Pad Software, Inc., San Diego, CA) and presented as mean ± SD of at least three independent experiments. Differences between two groups were evaluated using Student’s *t* test. *p* < 0.05 was considered to be statistically significant, *p* < 0.01 was considered to be highly statistically significant, *p* < 0.001 was considered to be extremely statistically significant.

## Results

### Expression of Cytokines in Mouse and Human Macrophages Induced by Lipopolysaccharide

**FIGURE 1:**
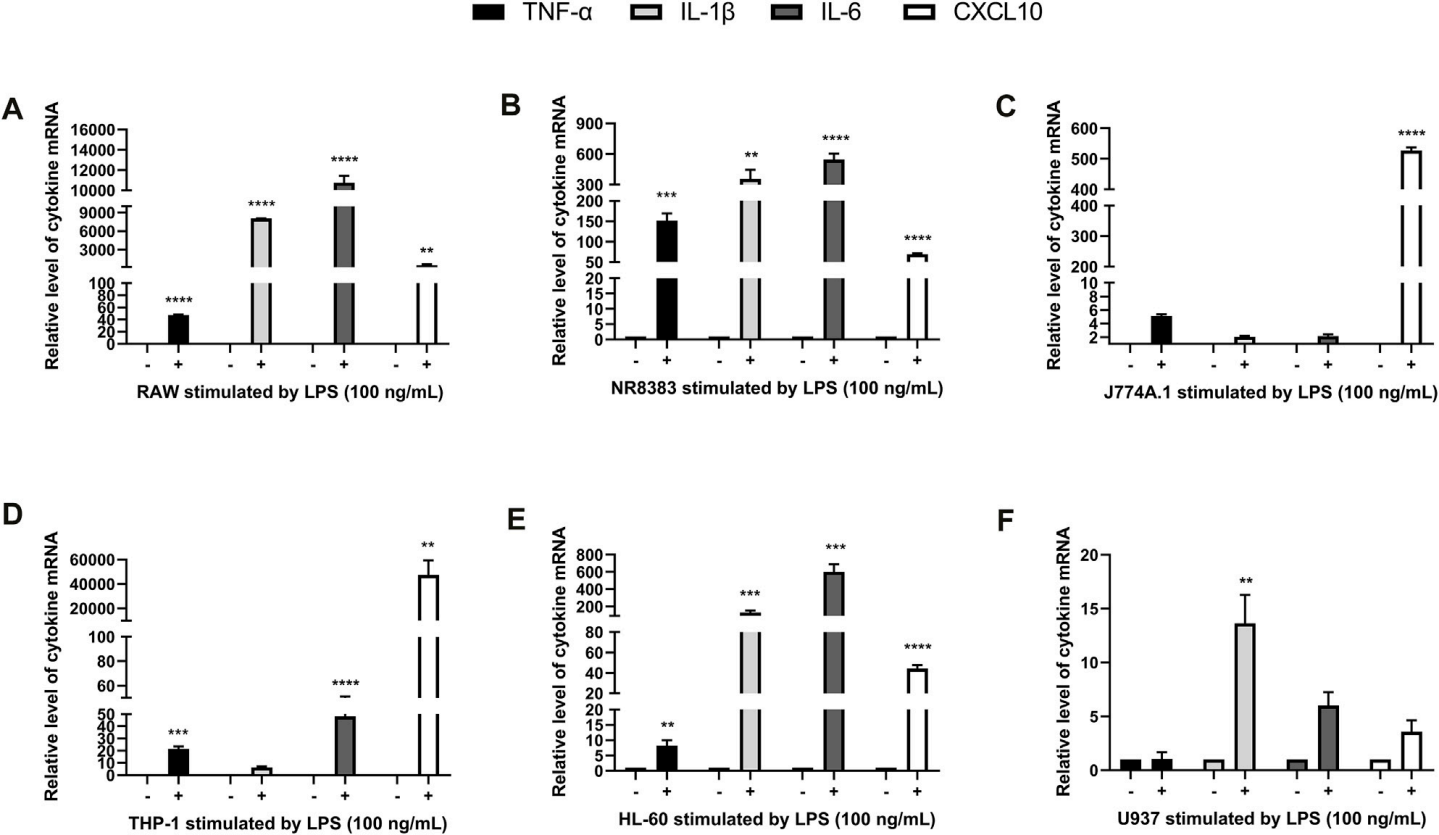
LPS stimulated cytokine expression in mouse and human macrophages. RT-PCR quantification of TNF-α, IL-6, IL-1β, and CXCL10 mRNA expression in RAW **(A)**, NR8383 **(B)**, J774A.1 **(C)**, THP-1 **(D)**, HL-60 **(E)** and U937 **(F)** cells stimulated with LPS. (mean ± SD, *n* = 3). **p < 0.01, ***p < 0.001, ****p < 0.0001 vs. Control group.

In order to observe the effect of LPS on the expression of different cytokines in mouse and human cells, we used LPS to induce RAW, NR8383, J774A.1, THP-1, U937, and HL-60 cells, and then RT-PCR to quantitatively analyze the expression of IL-1β, TNF-α, IL-6, and CXCL10. Preliminary analyses of cytokine expressions induced by LPS showed that the concentration of 100 ng/ml for 4 h was most significant (data not shown). For mouse RAW and NR8383 cells the mRNA expression levels of IL-1β, TNF-α, IL-6 and CXCL10 cytokines increased significantly after LPS stimulation (*p* < 0.01), especially IL-6 and IL-1β. However, in J774A.1 cells, except for CXCL10, the changes in other cytokines were not obvious (Figures 1A–C). For human THP-1 and HL-60 cells, the mRNA expression levels of the four cytokines all increased significantly after LPS stimulation (*p* < 0.01). But in U937 cells, the expression levels of various cytokines had almost no significant changes compared with the control (Figures 1D–F). The above data suggested that LPS induced a significant increase in cytokine levels in human and mouse macrophages, and the changes in cytokine levels in mouse cells were more obvious.

### Effects of Trichomicin, Ebosin, and 1487B on the Cytotoxicity of RAW, NR8383, HL-60, and THP-1 Cells

**FIGURE 2:**
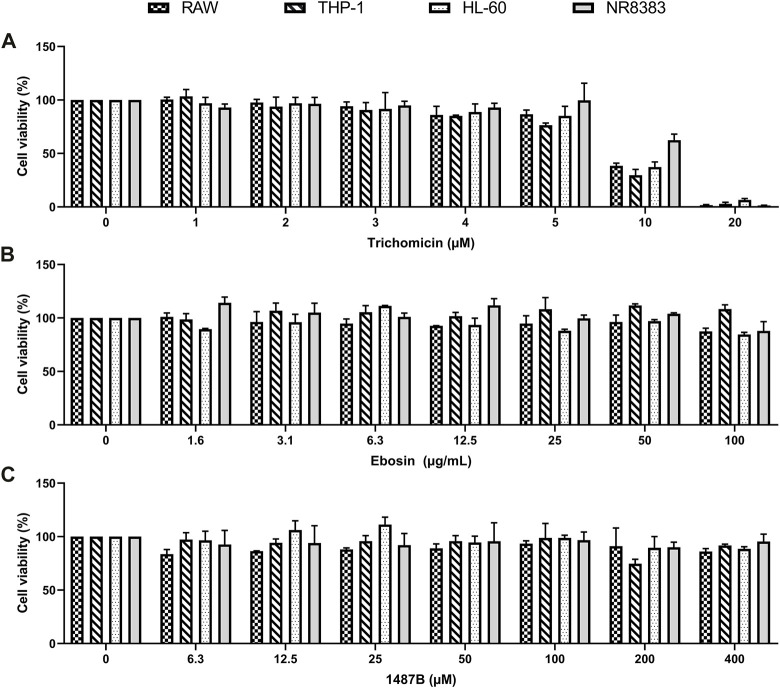
Effects of Trichomicin, Ebosin and 1487B on the viability of RAW, THP-1, NR8383, and HL-60 cells. **(A)** MTT assay of viability in the cells treatment with 1, 2, 3, 4, 5, 10, and 20 µM Trichomicin. **(B)** MTT assay of viability in the cells treatment with 1.6, 3.1, 6.3, 12.5, 25, 50, and 100 µg/ml Ebosin. **(C)** MTT assay of viability in the cells treatment with 6.3, 12.5, 25, 50, 100, 200, and 400 µM 1487B. (mean ± SD, *n* = 6).

To further explore the anti-cytokine storm activity of Trichomicin, Ebosin and 1487B, cell viability assay was performed. Cell lines sensitive to LPS induction, including Raw, NR8383, THP-1 and HL-60, were selected. The cells were cultured with Trichomicin (0, 1, 2, 3, 4, 5, 10, 20 μM), Ebosin (0, 1.6, 3.1, 6.3, 12.5, 25, 50, 100 μg/ml) and 1487B (0, 6.3, 12.5, 25, 50, 100, 200, 400 μM) for 72 h, and cell viability was determined by MTT assay. As shown in Figure 2, within the experimental concentrations, Ebosin and 1487B were almost non-toxic to the four cells. However, 20 µM of Trichomicin was toxic to cells, and 10 µM of Trichomicin was slightly toxic to cells, so the Trichomicin concentration in subsequent experiments should be lower than 10 µM.

### Cytokine Transcription Levels in RAW, NR8383, HL-60 and THP-1 Cells Were Inhibited by Trichomicin, Ebosin and 1487B

Cytokines such as IL-1β, TNF-α, IL-6, and CXCL10 are central players in CRS, thus we further investigated the effects of Trichomicin, Ebosin and 1487B on the expression of these cytokines in different macrophages. Based on the results induced by LPS (Figure 1), RAW, NR8383 mouse cells and THP-1, HL-60 human cells were selected for the experiment. According to the results of the cell viability assay (Figure 2), maximum concentrations of Trichomicin, Ebosin, and 1487B were 8 μM, 12,800 ng/ml and 400 μM, respectively. The cells were treated with Trichomicin, Ebosin, and 1487B for 30 min, and then stimulated with LPS for 4 h. Then RNA was extracted, and the expression of IL-1β, TNF-α, IL-6 and CXCL10 was quantitatively analyzed by RT-PCR after reverse transcription.

**FIGURE 3:**
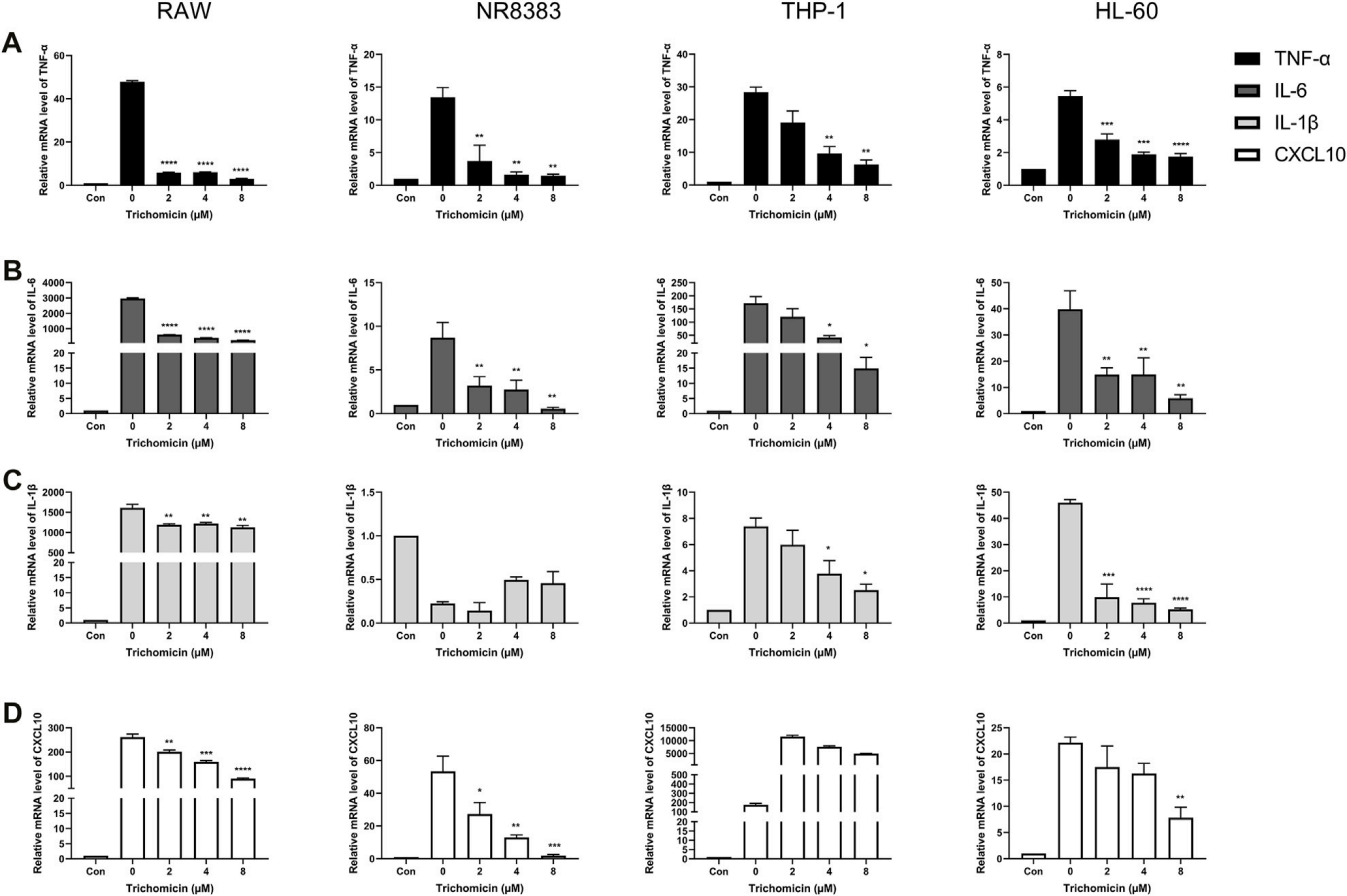
Trichomicin inhibits the expression of TNF-α, IL-6, IL-1β, and CXCL10 in macrophages. RT-PCR quantitation of TNF-α **(A)**, IL-6 **(B)**, IL-1β **(C)** and CXCL10 **(D)** mRNA expression in RAW, NR8383, THP-1, and HL-60 cells stimulated with LPS (100 ng/ml) following treatment with 2, 4, and 8 µM Trichomicin. (mean ± SD, *n* = 3). *p < 0.05, **p < 0.01, ***p < 0.001, ****p < 0.0001 vs. LPS-induced group.

**FIGURE 4:**
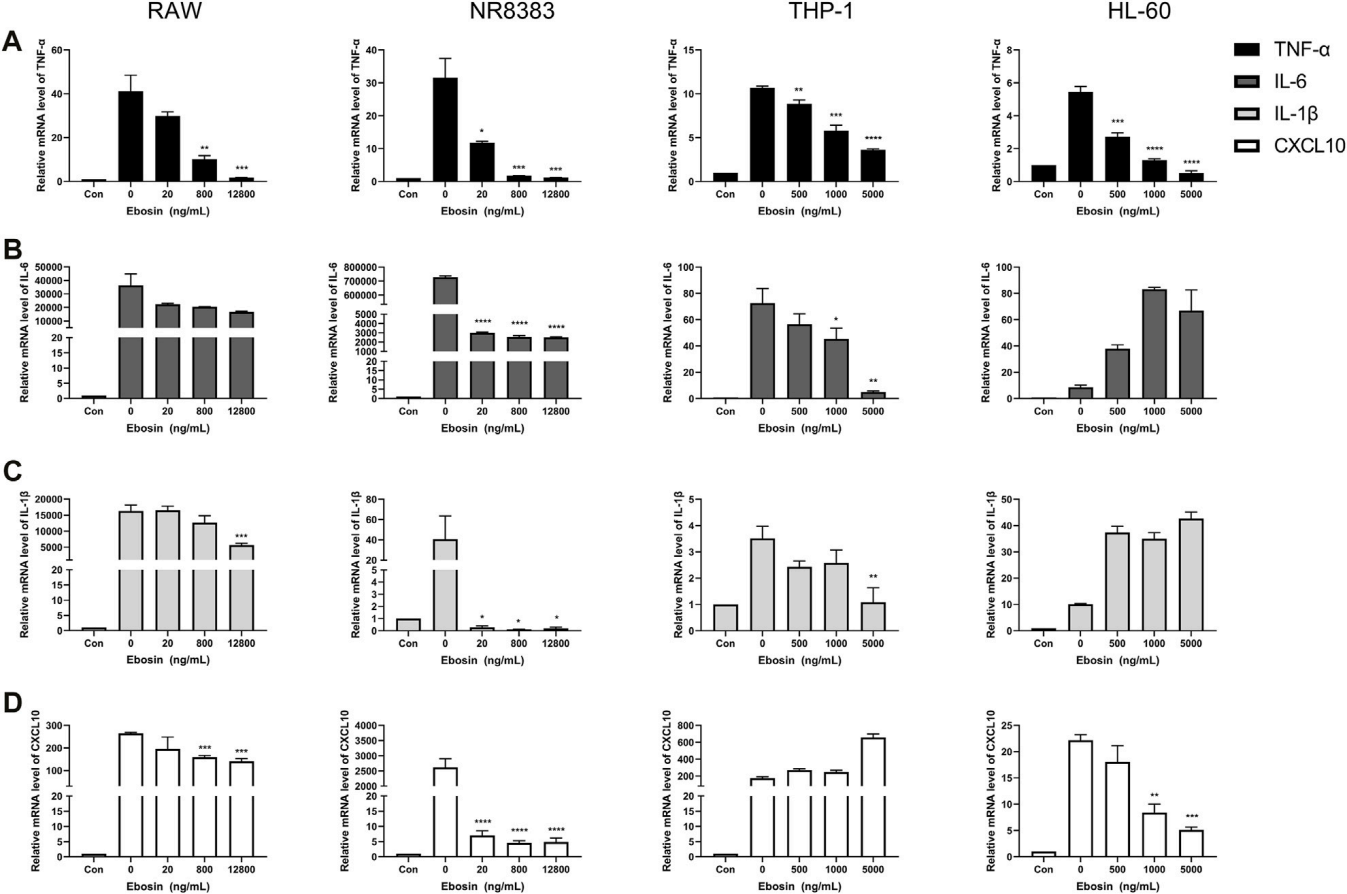
Ebosin inhibits the expression of TNF-α, IL-6, IL-1β, and CXCL10 in macrophages. RT-PCR quantitation of TNF-α **(A)**, IL-6 **(B)**, IL-1β **(C)** and CXCL10 **(D)** mRNA expression in RAW and NR8383 cells stimulated with LPS (100 ng/ml) following treatment with 20, 800, and 12,800 ng/ml Ebosin, and in THP-1 and HL-60 cells stimulated with LPS (100 ng/ml) following treatment with 500, 1,000, and 5,000 ng/ml Ebosin. (mean ± SD, *n* = 3) *p < 0.05, **p < 0.01, ***p < 0.001, ****p < 0.0001 vs. LPS-induced group.

**FIGURE 5:**
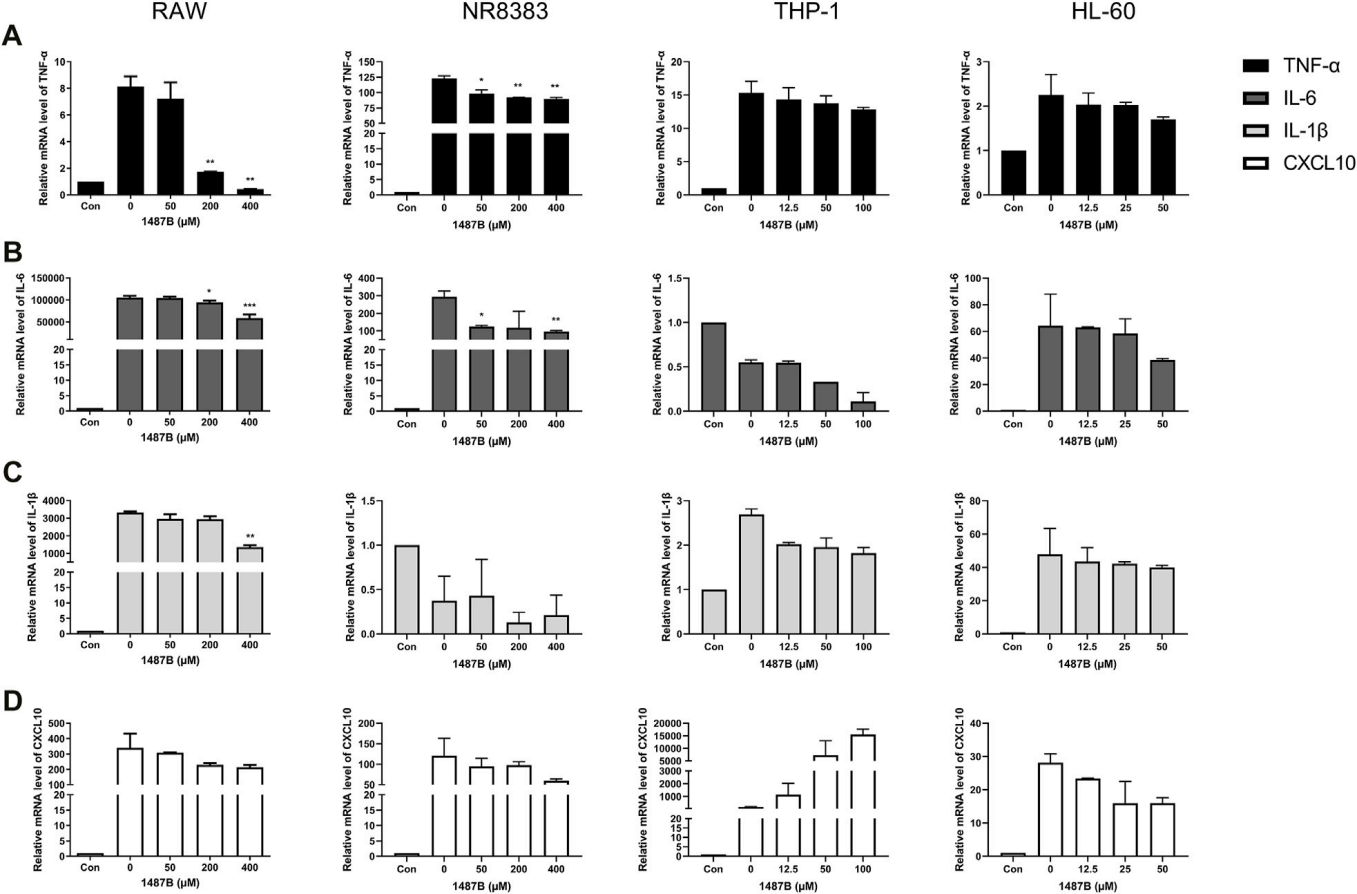
1487B inhibits the expression of TNF-α, IL-6, IL-1β, and CXCL10 in macrophages. RT-PCR quantitation of TNF-α **(A)**, IL-6 **(B)**, IL-1β **(C)** and CXCL10 **(D)** mRNA expression in RAW and NR8383 cells stimulated with LPS (100 ng/ml) following treatment with 50, 200, and 400 µM 1487B, and in THP-1 cell stimulated with LPS (100 ng/ml) following treatment with 12.5, 50, and 100 µM 1487B, and in HL-60 cell stimulated with LPS (100 ng/ml) following treatment with 12.5, 25, and 50 µM 1487B. (mean ± SD, *n* = 3) *p < 0.05, **p < 0.01, ***p < 0.001 vs. LPS-induced group.

As shown in Figure 3, Trichomicin had a significant inhibitory effect on the expression of TNF-α, IL-6, IL-1β and CXCL10 in a dose-dependent manner, with the exception of IL-1β in NR8383 and CXCL10 in THP-1. Among them, Trichomicin had the most significant inhibitory effect on the TNF-α and IL-6 factors of RAW and NR8383 mouse cells, especially RAW cells. In RAW cells stimulated with LPS, the expression of TNF-α mRNA was decreased to 6.0 ± 0.2 (*p* < 0.001), 5.9 ± 0.2 (*p* < 0.001), and 3.0 ± 0.5 (*p* < 0.001), and the expression of IL-6 mRNA was decreased to 601.9 ± 6.4 (*p* < 0.001), 401.5 ± 8.7 (*p* < 0.001), and 243.8 ± 11.5 (*p* < 0.001) in response to 2, 4, and 8 μM Trichomicin, respectively. Compared with RAW cells stimulated by LPS, the expression of TNF-α was reduced by 15.3 times and the expression of IL-6 was reduced by 11.2 times in cells treated with 8 μM of Trichomicin. Similarly, Ebosin had a significant inhibitory effect on the four factors in mouse cells, all of which are statistically significant (Figure 4). Obviously, the IL-6 expression level in NR8383 cells treated with the maximum concentration of Ebosin was 482.6 times lower than cells treated with LPS (Figure 4B). Furthermore, the expression levels of TNF-α and IL-6 in all cells tend to decrease with the increase of 1487B concentration, but not significant (Figure 5).

In summary, the three drugs had obvious inhibitory effects on the expression of TNF-α and IL-6 in all cells, of which Trichomicin was the most significant. It was also found that the three drugs inhibited the expression of cytokines in mouse cells in a more significant manner. Therefore, we believe that the three drugs have inhibitory activity on the cytokine storm caused by LPS, of which Trichomicin is the one with best activity.

### Effect of Trichomicin on Mortality and Weight Changes in a Mouse Model

**FIGURE 6:**
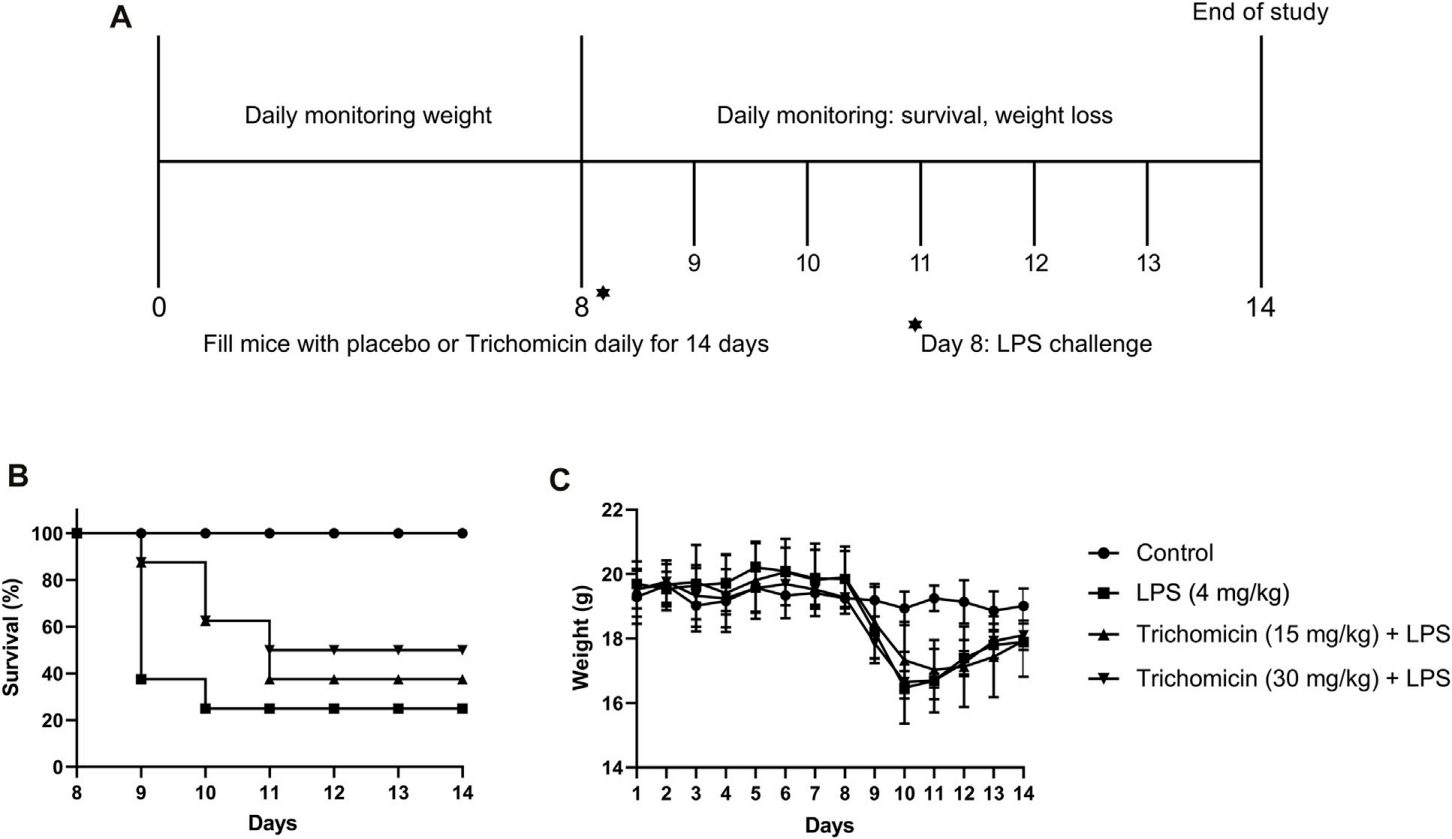
Trichomicin protected mice from LPS challenge. **(A)** Overview of studies investigating the use of Trichomicin as a therapeutic for LPS infection. BALB/c mice were challenged with 4 mg/kg of LPS. Mice received a 14-day’s intragastrically treatment of Trichomicin or placebo. **(B)** Survival rate within the 14 days’ study. **(C)** Weight changes during the 14 days.

According to the results of the RT-PCR experiment, the three drugs inhibited various cytokines in mouse cells, and the inhibition of Trichomicin was the most obvious. We further explored the inhibitory activity of Trichomicin *in vivo*. After preliminary dose exploration, 4 mg/kg LPS was used for modeling, and Trichomicin at 15 and 30 mg/kg were used in the low-dose and high-dose treatment groups. As shown in Figure 6, both low-dose and high-dose of Trichomicin reduced the mortality of mice compared with the control, and high-dose of Trichomicin was more effective. In addition, following treatment with LPS, the body weight of mice within the model and Trichomicin treatment groups were significantly lower than that of the blank control, and their body weight were gradually recovered after 3 days of treatment.

### Trichomicin Downregulates Lipopolysaccharide–Induced NF-κB Pathway Activation and Blocks Basal Stat3 Phosphorylation in Macrophages

**FIGURE 7:**
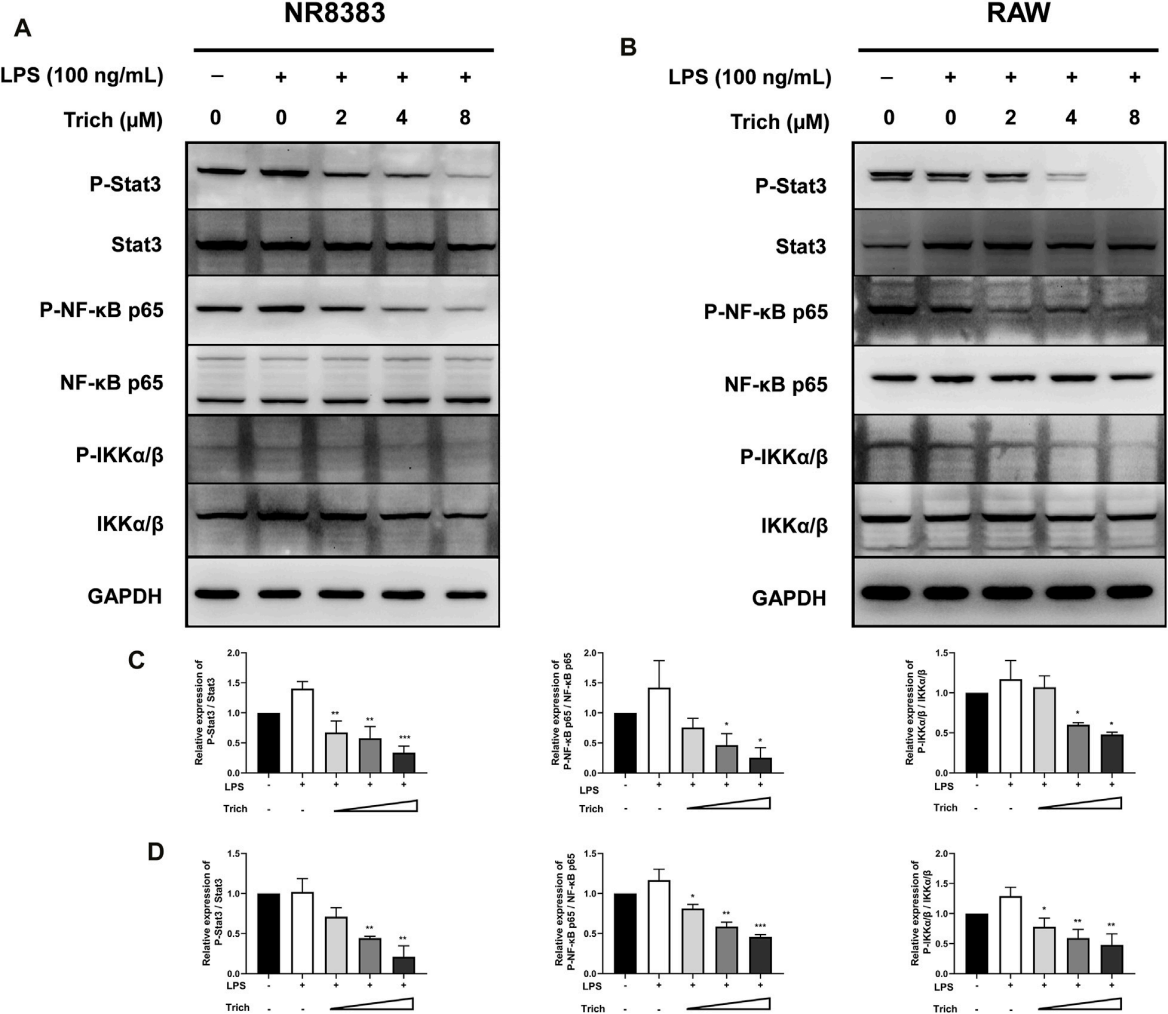
Western blot analysis of LPS-induced NF-κB p65, IKKα/β and Stat3 phosphorylation in NR8383 **(A,C)** and RAW **(B,D)** cells treated with Trichomicin. (mean ± SD, *n* = 3). *p < 0.05, **p < 0.01, ***p < 0.001 vs. LPS-induced group.

Macrophages have historically been considered to be the main source of IL-6 and TNF-α. To further elucidate the role of Trichomicin in the CRS, we evaluated signaling transduction pathways associated with TNF-α and IL-6 expression in macrophages by immunoblotting. Due to the fact that Trichomicin inhibited the expression of cytokines in mouse cells more significantly, we decided to use RAW and NR8383 mouse cells in this assay. Followed by a 4 h’s stimulation with LPS, macrophages were preincubated with various concentrations of Trichomicin for 30 min, and then the phosphorylation of NF-κB p65, IKKα/β and Stat3 were analyzed. The results showed that phosphorylation of Stat3 and phosphorylation of NF-κB p65 were significantly inhibited by treatment with 4 and 8 μM Trichomicin (*p* < 0.05) (Figure 7). It can be concluded that Trichomicin inhibited the NF-κB and Stat3 pathways, and then inhibited the response of macrophages to pro-inflammatory stimuli.

## Discussion

The cause of cytokine storm is generally considered to be the overreaction of immune system to new and highly pathogenic pathogens, that is, the imbalance of immune regulatory network. The lack of negative feedback and the continuous self-amplification of positive feedback make a variety of cytokines abnormally increasing, eventually leading to single or multiple organ damage, functional failure and death. As early as 1989, in the clinical application of the anti-T cell antibody OKT3, it was discovered that following the first treatment, patients would have a series of uncomfortable symptoms such as elevated body temperature, headache, nausea, and releasement of a large number cytokines. These symptoms will be relieved following glucocorticoid treatment, and the concept of CRS has also been formally put forward (Chatenoud et al., 1989). The CRS caused by SARS infection in 2003 would cause multiple organ failures, resulting in extremely high mortality rates, thus has attracted more attention (de Jong et al., 2006). This COVID-19 epidemic has pushed the research of CRS to a climax, making it a key research direction.

When the body is infected or traumatized, its immune system produces immune response to eliminate or destroy antigens. When a pathogen invades the body, the innate immune system first takes effect. Epithelial cells infected by the pathogen produce a small amount of cytokines such as IFN-α/β, IL-1β, IL-8, etc. NK cells stimulated by IFN-α/β release a small amount of INF-γ to activate macrophages, and activated macrophages release large amounts of IL-6, TNF-α, IL-12 and other cytokines, which in turn activate NK cells. Therefore, “positive feedback” is formed between NK cells and macrophages, and cytokines being released increase drastically (Wang et al., 2018b). At the same time, pathogens are processed by the innate immune system and are recognized as antigens, and the adaptive immune system composed of B cells and T cells begins to play a role. A large amount of IL-12 and IFN-γ cytokines released by T-helper-1 (Th1) cells not only stimulate their own division and proliferation, but also stimulate the activation of macrophages to further activate the innate immune system, which can also generate “positive feedback” (Shimabukuro-Vornhagen et al., 2018). Normally, once the body controls the invading pathogen under the regulation of “positive feedback,” the signal presented by the antigen to the adaptive immune system is weakened, the release of cytokines begins to decrease, and the inflammatory response gradually gets weakened, resulting in “negative feedback.” Moreover, there are some inhibitory cytokines in the immune system, such as IL-10, TGF-β, and sphingosine 1-phosphate (S1P), which act on vascular endothelial cells to regulate excessive immune response and carry out “negative feedback” regulation on the body (Tisoncik et al., 2012). However, when the body is attacked by a violent virus such as COVID-19, the human immune system will release a large amount of cytokines under the action of “positive feedback,” and the cytokine signals will be extremely amplified. The body’s “negative feedback” regulation is too weak and too late, which will lead to an imbalance in the body’s immune regulatory network, causing a CRS and worsening the disease.

LPS enters the cell by binding to receptors such as CD14 and Toll-like receptor 4 (TLR4) located on the membrane of monocytes/macrophages. A series of enzymatic reactions are activated through signal transduction pathways to promote activation or translocation of transcription factors into the nucleus, thereby regulate the expression of many genes (Zhang and Cao, 2003). Specifically, TLR4 activates host defenses by rapidly triggering the inflammatory response in the LPS recognition pathway, transferring to CD14 and presenting LPS to the TLR4-MD2 complex, thereby dimerizes from the plasma membrane and initiates the TIRAP-MyD88-dependent pathway, leading to NF-κB activation. NF-κB activation is essential for maintaining NF-κB-dependent transcription of genes encoding inflammatory cytokine, especially in macrophages (Granucci, 2018). Subsequently, it induces the phosphorylation of nuclear transcription factor-κB (NF-κB) p65 protein and its IKKα in the Stat3 signaling pathway, leading to the production of tumor necrosis factor (TNF)-α, interleukin (IL-1β and IL-6) cytokines and chemokines (Zusso et al., 2019). Being injected with LPS, most tissues and organs can be activated to produce pro-inflammatory factors, and monocytes/macrophages are the main secreting cells of these factors. For example, rats were injected with LPS or *E. coli*, and *in situ* analysis showed that a large amount of IL-α and IL-β were produced in the spleen (Ge et al., 1997). The bone marrow mononuclear cells of mice treated with endotoxin showed overexpression of TNF-α (Schmauder-Chock et al., 1994). Chensue et al. found that in the LPS-induced sepsis mouse model, liver Kupffer cells were a major source of TNF and IL-1 production (Chensue et al., 1991). In this study, it was confirmed that after macrophages were stimulated by LPS, RT-PCR showed that the mRNA expression of TNF-α, IL-1β, IL-6 and CXCL10 in the cells was increased.

Other cytokines, such as MIP-1 and CRP, which also are the major underlying factors of CRS. CCL3 (MIP-1alpha), CCL4 (MIP-1beta), CCL9/10 (MIP-1delta) and CCL15 (MIP-1gamma) were produced by many cells, especially macrophages, dendritic cells and lymphocytes. MIP-1 proteins, which act via G-protein-coupled cell surface receptors (CCR1, 3, 5), expressed by lymphocytes and monocytes/macrophages, are best known for their chemotactic and proinflammatory effects but can also promote homoeostasis. Moreover, CRP, ferritin, and procalcitonin are sensitive markers of COVID-19 disease in the acute phase. The elevated CRP level of COVID-19 patients is strongly correlated with disease severity and prognosis. The severity of COVID-19 can be assessed by detecting inflammatory biomarkers, including high levels of IL-6 and plasma CRP, and the release of these factors is closely related to ARDS. In addition, high levels of CRP and procalcitonin in the serum are also the main factors for poor prognosis.

Our study proves that Trichomicin, Ebosin and 1487B inhibited the expression of TNF-α, IL-1β, IL-6 and CXCL10 factors in mouse macrophages in varying degrees, while Trichomicin is the most effective one (Figures 3–5). Subsequent western blot experiments found that Trichomicin inhibited the Stat3 and NF-κB pathways and reduced the phosphorylation of Stat3 and p65 following LPS stimulation (Figure 7). Through comparison, the anti-cytokine storm activity of Trichomicin was significantly stronger than that of entecavir (Su et al., 2020). Trichomicin also showed good oral absorption and rapid uptake (Zhu et al., 2020). In addition, Scarneo et al. have proved that the new TAK1 inhibitor Takinib could inhibit the response of macrophages to pro-inflammatory stimuli. Also, Takinib reduced the phosphorylation of downstream proteins p-38, c-Jun, and NF-κB following LPS stimulation (Scarneo et al., 2018). Liu et al. established a U937 cell model based on human monocytes for high-throughput anti-influenza drug screening. Expression of three cytokine indicators CCL2, CXCL10 and viral NA were selected to screen immunomodulators and antiviral agents for the treatment of influenza. It was verified that at cellular level, the NF-κB pathway mainly regulates the expression of IL-6, IL-8, TNF-α, CCL2, CCL3, and CCL5 (Liu et al., 2019). On the other hand, *in vivo* studies have found that Clopidogrel, Sarpogrelate and Cilostazol all had inhibitory effects on the expression of TNF mRNA in the blood after LPS stimulation. Further study of the mechanism found that Cilostazol attenuated TNF-mediated phosphorylation of MAPKs and NF-κB p65 (Lee et al., 2020). LMT-28 could alleviate arthritis and acute pancreatitis in mice, reduce the secretion of TNF-α in serum, and inhibit IL-6-induced phosphorylation of Stat3, gp130 and JAK2 proteins (Hong et al., 2015). The above findings corroborate the results of this article, i.e., the phosphorylation of downstream Stat3 and p65 following LPS stimulation can be reduced by agents, eventually inhibits CRS. Based on these results, two indicators of IL-6 and TNF-α have been selected to establish a RAW cell model for the screening of anti-cytokine storm activity. The results of the three drugs purified in our laboratory show that Trichomicin has the best activity.

In summary, we used RT-PCR to detect the expression of representative cytokines in mouse and human cells treated with different concentrations of Trichomicin, Ebosin and 1487B following LPS stimulation. The results showed that the expression of TNF-α, IL-1β, IL-6, and CXCL10 in both cell lines increased after LPS stimulation, and the three drugs inhibited the expression of various factors to varying degrees. Trichomicin had the most obvious inhibitory activity on the expression of cytokines, and the change of cytokine expression is more significant in mouse cells. We subsequently verified that Trichomicin can improve the survival rate of mice treated with LPS, and the survival rate of high-dose group is higher. Furthermore, our research on the mechanism of Trichomicin inhibiting cytokine expression in mouse cells showed that Trichomicin inhibited the Stat3 and NF-κB pathways, thereby inhibiting the response of macrophages to pro-inflammatory stimuli.

The article clarifies that the inhibitory activity of the three drugs on CRS and its mechanism of action have important scientific significance and application value, which lays a foundation for the screening of anti-cytokine storm activity from microbial natural products. Although it was reported that blocking cytokine signaling may impair clearance of influenza virus, increase the risk of secondary infections, and lead to worse outcomes (Koch et al., 2018), until now we found no reports that blocking cytokine signaling may impair clearance of SARS-CoV-2. Trichomicin has the potential to be developed as a treatment for COVID-19, however, we will maintain sustained attention about secondary infections of SARS-CoV-2 after blocking cytokine signaling.

## Data Availability

The original contributions presented in the study are included in the article/Supplementary Material, further inquiries can be directed to the corresponding authors.
